# The Role of Agreeableness, Neuroticism, and Relationship-Specific Features in Self- and Other-Perceptions of Conflict Frequency in Adolescent Relationships with Parents and Peers

**DOI:** 10.1007/s10964-024-01951-6

**Published:** 2024-02-24

**Authors:** Eva Bleckmann, Larissa L. Wieczorek, Jenny Wagner

**Affiliations:** Department of Educational Psychology and Personality Development, Institute of Psychology, University of Hamburg, Hamburg, Germany

**Keywords:** Adolescence, Conflict, Personality, Social relationships, Multi-rater perspectives

## Abstract

Conflict frequency in adolescence has been linked to personality and relationship-specific features. However, an integrative investigation of both aspects is lacking. To address this gap, this study used data from 571 individuals in middle adolescence (Study 1; *M*age = 15.86, SD = 1.23; 75.8% female) and 233 individuals in late adolescence (Study 2; *M*age = 17.17, SD = 1.01; 75.5% female) in Germany, including participants’ self-reports on conflict frequency and other-reports provided by parents and peers. Across studies, multigroup models revealed that adolescents’ level of neuroticism predicted self- and other-perceived conflict frequency in parent and peer relationships more consistently than agreeableness, while providing no evidence for an interplay between both personality traits. Furthermore, relationship-specific features differentially accounted for individual differences in conflict frequency across relationship types, such that in adolescents’ relationships with parents, lower relationship quality related to more frequent conflicts. In peer relationships, higher contact frequency was linked to more frequent conflicts. The present findings highlight the contributions of both personality and relationship-specific features to conflict frequency in adolescence and offer practical guidance for the improvement of adolescents’ and their relationship partners’ social skills and experiences. All research questions, hypotheses, and analyses of this research were preregistered at the OSF and can be retrieved from: https://osf.io/xmvqd/.

## Introduction

In adolescence, conflicts take on a special significance. Social networks and relationships change significantly during this period (e.g., Wagner et al., 2014) and interpersonal conflicts occur more frequently than in other phases of life (e.g., Smetana et al., 2006). These changes offer a unique possibility for adolescent psychosocial development, as conflicts promote an individual’s understanding of other people’s feelings and intentions, their own needs, and the social rules and conventions that guide interpersonal behavior (Dunn & Slomkowski, 1992). Accordingly, conflicts are a normative aspect of the adolescent transition from childhood to adulthood (Branje, 2018). At the same time, frequent arguments can be detrimental to any type of relationship, affecting the well-being and mental health of those involved (Ehrlich et al., 2012; Laursen & Hafen, 2010). One key predictor of conflict frequency is an individual’s personality, that is, the way people tend to think, feel, and behave across situations (Roberts et al., 2006). In particular, low levels of agreeableness and high levels of neuroticism have been associated with a higher number of interpersonal conflicts (e.g., Deventer et al., 2019). However, most findings on personality and conflict frequency are based on research with adults (e.g., Berry et al., 2000; Mund & Neyer, 2014). It therefore remains an open question if results generalize to adolescents. Another key ingredient of conflict frequency concerns the relationship features that characterize the social bond between two people. Specifically, relationships vary in their functions and in their unique characteristics, and these differences affect how frequently conflict emerges (Canary et al., 1995). Bridging previous findings from personality psychology and developmental research, the current study investigated how adolescents’ personality (i.e., agreeableness and neuroticism) and relationship features (i.e., contact frequency and relationship quality) are related to conflict frequency in adolescent parent and peer relationships. To achieve a comprehensive understanding of these links, a multi-rater approach was adopted and both adolescents’ self-perceptions of conflict frequency and other-perceptions provided by adolescents’ parents and peers were considered.

### Personality and Conflict Frequency: The Role of Agreeableness and Neuroticism

Previous research indicates that individuals scoring high on agreeableness are motivated to cooperate and get along with others (Denissen & Penke, 2008a). They possess strong self-regulatory capabilities to control negative thoughts or behavioral impulses in social situations (Robinson, 2007). Accordingly, people with higher agreeableness are less likely to initiate conflicts and adapt more efficiently to their relationship partners’ behaviors, which reduces the risk of conflicts. Despite these strong behavioral implications, findings regarding agreeableness and conflict frequency are mixed. Among the existing studies on this topic, which are exclusively based on samples from Westernized countries, some reported that high levels of agreeableness are linked to less self-reported conflict (e.g., Bono et al., 2002), while others did not find a significant association with self- or other-perceived conflict measures (e.g., Jensen-Campbell et al., 2003).

Regarding neuroticism, theoretical accounts and empirical findings suggest that individuals with high trait levels tend to feel anxious and easily stressed (e.g., Barlow et al., 2014). They process information in a more negative way (Finn et al., 2013) and show an increased sensitivity to negative social signals (Denissen & Penke, 2008b). Accordingly, people with higher neuroticism likely interpret their social relationships more negatively and perceive them as more conflictual. In line with these implications for cognitive processing, empirical evidence illustrates a strong link between a person’s level of neuroticism and their self-perceived conflict frequency (e.g., Mund & Neyer, 2014). In addition, poorer problem-solving skills and less control of behavioral impulses (e.g., Shiner, 2019) might also provoke more conflict from the partner’s perspective. Indeed, several studies focusing on adult samples have demonstrated that high neuroticism levels are linked to higher rates of other-perceived conflict (e.g., Berry et al., 2000).

Finally, the interplay of both traits might predict perceptions of conflict frequency across relationships beyond the main effects of agreeableness and neuroticism: Both theoretical (Hofstee et al., 1992) and empirical (Ode et al., 2008) notions suggest that the implications of a person’s agreeableness for interpersonal behavior can differ depending on their level of neuroticism and vice versa. Building on this, there are two alternative hypotheses about the interplay of agreeableness and neuroticism in relation to conflict frequency. First, the combination of high levels of agreeableness and neuroticism could lead to a *mutual attenuation effect*: The self-regulatory abilities associated with agreeableness (Robinson, 2007) may help to control the impulsive behaviors and strong emotional reactions associated with neuroticism, while the negative perceptions associated with neuroticism (e.g., Finn et al., 2013) may interfere with self-regulatory behaviors associated with agreeableness (Ode et al., 2008). Second, the interplay of high levels of agreeableness and neuroticism could lead to a *sensitization effect*: Individuals high on both agreeableness and neuroticism may be more sensitive to social cues (see Hofstee et al., 1992) and particularly alert to potential conflicts in their social relationships (Denissen & Penke, 2008a), while also possessing the skills to navigate complex social situations (Robinson, 2007). This way, they might be more successful at preventing conflict in the first place and thus perceive even lower levels of conflict frequency.^1^

Taken together, agreeableness has been associated with self- and other-perceptions of lower conflict frequency to some extent, whereas neuroticism has been robustly associated with self- and other-perceptions of higher conflict frequency. Moreover, both personality traits might interact and either attenuate each other’s effects or lead to a sensitization for social dynamics that helps to prevent conflict. So far, most findings on personality and conflict frequency are based on research with adult samples. In adolescence, however, existing literature on personality development highlights temporary decreases in agreeableness and increases in neuroticism compared to childhood and later adulthood. This phenomenon is often referred to as the *disruption hypothesis* (e.g., van den Akker et al., 2014). Age-specific changes in personality may introduce age-sensitive social dynamics (e.g., less agreeable behavior) that could contribute to higher conflict frequency in adolescence (see Smetana et al., 2006). Therefore, examining the role of personality in conflict frequency during the developmental phase of adolescence is particularly important.

### Relationship-Specific Features and Conflict Frequency: Differences in Adolescent Parent and Peer Relationships

Conflict is an essential form of communication, serving as a signal for the importance of certain topics and as a catalyst for personal development and relationship transformation in adolescence (Adams & Laursen, 2007). One relationship marked by increased conflict frequency is that between adolescents and their parents (Branje, 2018). In adolescence, individuals seek to become autonomous and move towards more equal relationships with their parents, while still depending on them emotionally and financially. Whereas high conflict frequency in parent relationships has been related to lower relationship quality and maladjustment (Laursen & Collins, 1994), research consents that conflict with parents contributes to a functional transformation of family relationships (e.g., Adams & Laursen, 2001). Moreover, research illustrates that conflicts with parents predominantly focus on responsibilities, autonomy, or school, and adolescents engage in conflict behaviors aimed at asserting their interests rather than maintaining an amicable relationship (Laursen, 1995). Thus, although parents remain central attachment figures for adolescents (Smetana et al., 2006), the relationship is characterized by ambivalence (see Fingerman et al., 2008).

A second important type of relationship in adolescence is with peers, which includes relationships with close friends, romantic partners, clique members, or other acquaintances with people of the same age outside the family (Collins & Steinberg, 2006). Despite differences in their specific function and degree of closeness, relationships with peers have in common that they are voluntary, which stands in contrast to relationships with parents (Laursen & Pursell, 2009). Peer relationships are driven by the desire to expand one’s social network and to build meaningful connections outside the family (De Goede et al., 2009). Thus, although parent and peer relationships share functions, such as providing adolescents with company and emotional as well as practical support, their meaning for adolescents’ psychosocial development also differs in important aspects and shifts with time (Laursen & Bukowski, 1997). Over the course of adolescence, closeness with peers increases and often exceeds the closeness in family relationships (Adams & Laursen, 2007). Along with this relational development, conflicts in peer relationships mainly pertain to interpersonal aspects, and adolescents typically select conflict strategies that minimize the likelihood of relationship dissolution or other disruptive effects of dispute (Laursen, 1995). This way, peer relationships offer opportunities to try out new strategies to improve social skills, including conflict management (Scholte & Van Aken, 2006).

Together, parents and peers represent significant relationship partners in adolescence, each taking on different functions in adolescent psychosocial development. Correspondingly, conflict in parent and peer relationships typically differs with respect to discussed topics and with respect to the approaches which adolescents take to deal with them. To understand the factors contributing to individual differences in the perceived frequency of conflicts, it is important to distinguish between conflicts across relationship types. Moreover, the cultural macrosystem in which adolescents’ conflicts with parents and peers occur needs to be considered (Bronfenbrenner & Morris, 1998). Besides culture-specific social norms regarding the behavior of adolescents and their relationship partners (e.g., Bornstein & Cheah, 2005), this also includes the historical context. One event that had a great impact on adolescents’ social lives was the COVID-19 pandemic. As part of the measures to contain the virus, interactions with peers were severely restricted, while time spent at home with family increased, challenging adolescents’ needs for autonomy and relationships outside the family context (Campione-Barr et al., 2021). A study from the US found that adolescents’ perceptions of conflict with family and peers decreased over the course of the pandemic on average, although there were considerable individual differences (Rogers et al., 2021). In sum, acknowledging the cultural and historical background is important for the investigation of the interplay of personality, relationship variables, and conflict.

Adding to the differences between adolescents’ relationships with parent and peers, conflicts also differ within specific relationship types depending on the unique features of each relationship. Specifically, the probability of conflict increases as relationships get closer, as there is more need to negotiate each other’s rights, responsibilities, and mutual assessments (Canary et al., 1995). On the other hand, previous research highlights that conflict occurs more frequently in relationships that are marked by lower quality and higher negativity (Adams & Laursen, 2007). Therefore, the frequency of contact between relationship partners and the perceived quality of a relationship both need to be considered when examining conflict frequency in adolescent parents and peer relationships. Furthermore, empirical research with samples of young adults indicates that self-perceptions and partner perceptions of closeness and conflict differ substantially (Berry et al., 2000). Moreover, studies show that adolescents’ perceptions of the family environment (Human et al., 2016), conflict intensity (Mastrotheodoros et al., 2020), or parental warmth (Janssen et al., 2021) can differ markedly from their parents’ perceptions. Similar discrepancies have been found in adolescent peer relationships focusing on adolescents’ and peers’ perceptions of relationship quality (Spilt et al., 2015) or bullying experiences (Hwang et al., 2017). Accordingly, both adolescents’ self-perceptions as well as parent and peer perceptions of conflict frequency were considered in the current study.

## Current Study

Although previous research has shown the key roles of personality and relationship-specific features for conflict frequency among adolescents, an integrative examination of both features is missing. Addressing this gap, the current research includes two studies investigating how the personality traits agreeableness and neuroticism relate to perceptions of conflict frequency in middle (Study 1) and late adolescence (Study 2). These associations are examined with regard to self- and other-perceptions of conflict frequency in adolescents’ relationships with parents and peers and accounting for the relationship quality and contact frequency within each relationship. It was expected that higher agreeableness is associated with self- and other-perceptions of lower conflict frequency (Hypothesis 1). Conversely, it was assumed that higher neuroticism relates to self- and other-perceptions of higher conflict frequency (Hypothesis 2). Regarding the interplay of both traits, there were two competing hypotheses: On the one hand, there may be a mutual attenuation effect, with higher levels of agreeableness and higher levels of neuroticism attenuating each other’s effects on self- and other-perceptions of conflict frequency (Hypothesis 3a). On the other hand, there may be a sensitization effect, with higher levels of both traits relating to self- and other-perceptions of lower conflict frequency (Hypothesis 3b). Both Studies 1 and 2 examined these hypotheses in relationships with parents and peers, considering self- and other-perceptions of conflict frequency as well as relationship-specific features (i.e., contact frequency and relationship quality). Given the lack of previous research in this area, there were no specific predictions with respect to the two relationship types; differences between parent and peer relationships were addressed as an exploratory third research question.

## Method

The present research was preregistered (https://osf.io/xmvqd/). Study 1 included the combined data of two samples focusing predominantly on middle adolescence (*M*_age_ = 15.86). The first subsample (*n* = 315) originated from a research project tracking students during the COVID-19 pandemic in November-December 2020 (Wagner et al., 2022; https://osf.io/r5gjx/), while the second subsample (*n* = 256) was collected during later pandemic phases in May-July 2021 (Bleckmann & Wagner, 2021; https://osf.io/w4nmj/). The two subsamples were merged to enhance statistical power. Study 2 was based on data from participants in late adolescence (*M*_age_ = 17.71) who were in their final year of high school and tracked over the course of their graduation (*n* = 233; Wagner et al., 2021; https://osf.io/4gnz9/). The data from Study 1 and Study 2 were analyzed separately for two reasons. First, the age distribution varied within the respective samples: Study 1 focused on participants aged mostly around 16 (*Mdn* = 16; range = 14–18), while Study 2 predominantly included older adolescents (*Mdn* = 18, range = 17–19). Second, both subsamples in Study 1 were collected during the COVID pandemic, whereas Study 2 was conducted in non-pandemic times. Table OS 1 in the Online Supplement (OS) displays an overview of the number of self- and other-reports in Study 1 and Study 2, Table OS 2 includes an overview of the self- and other-reports in the subsamples of Study 1.

### Participants

Table 1 shows the demographic information for participants in Study 1 and Study 2. In total, Study 1 contained data from 571 adolescents and Study 2 contained data from 233 adolescents.^2^ In both studies, adolescents provided up to five self-reports regarding their relationships with up to five close others. Study 1 contained *n* = 246 self-reports on social relationships with parents and *n* = 525 reports on relationships with peers. Study 2 included *n* = 196 self-reports on relationships with parents and *n* = 483 on self-reports on peer relationships. Next, adolescent participants were asked to invite up to five people to fill out other-reports regarding their relationship with the adolescent participant. Regarding the informants who filled out these other-reports, Study 1 included *n* = 219 parent reports (*M*_age_ = 46.63, 68.49% female) and *n* = 407 reports from peers (*M*_age_ = 16.48, 73.96% female). Study 2 included 70 reports from parents (*M*_age_ = 50.39, 71.23% female) and 161 reports from peers (*M*_age_ = 17.96, 65.56% female).

**Table 1:** Demographic information of adolescent participants in Study 1 and Study 2

Variables	Study 1 (*n* = 571)	Study 2 (*n* = 233)
Age (*M*, SD)	15.86 (1.23)	17.71 (1.01)
Gender (% female)	75.83%	75.54%
Attending high school	78.47%	100%
German as first language (% yes)	91.94%	88.84%
Born in Germany (% yes)	94.73%	94.42%
Living with parents	92.01%	93.56%
Education father		
No school leaving certificate	1.22%	3.86%
High school diploma or equivalent	45.83%	45.92%
University degree	33.85%	34.33%
Not specified	19.10%	15.89%
Education mother		
No school leaving certificate	2.10%	3.43%
High school diploma or equivalent	52.10%	46.78%
University degree	28.99%	36.91%
Not specified	16.81%	12.88%

Education of fathers and mothers was assessed as an indicator of socioeconomic status

The power for multiple regression models for self- and other-reported conflict frequency was estimated in Study 1 and Study 2 prior to the analyses. Results suggested that an assumed overall sample size of *n* = 738 self-reports and *n* = 634 other-reports in Study 1 provided sufficient power ( < 0.80) to detect small effects (*f*^2^ < 0.024) in a model with eight predictor variables. Similarly, an assumed sample size of *n* = 679 self-reports and *n* = 235 other-reports in Study 2 suggested sufficient power for detecting small effects (*f*^2^ = 0.021 and *f*^2^ = 0.063).

### Missing Data

Within each study, there was no missing data, since the study design featured mandatory answers on each item. However, there were differences in data availability across studies. As displayed in Table OS 1, Subsample 2 in Study 1 (*n* = 256 adolescents) did not include self-reports on relationship-specific variables by design. Consequently, self-report data on relationships was not available for these participants. In the reports provided by relationship partners (i.e., other-reports), again by design, the partners were not asked to specify their relationship type with the target adolescent. Therefore, the categories “parents” and “peers” were constructed using theoretically derived and empirically tested criteria (e.g., age difference, relationship duration). The full procedure and validation of the reconstructed relationship types are detailed on the OSF (https://osf.io/xmvqd/).

### Procedure

Participants of both studies were recruited via social media and schools. Ethical approval for data collection of Study 1 and Study 2 was granted by the local ethics committee of the University of Hamburg and by the German Psychological Society (DGPs), respectively. The procedure of both studies was similar: First, participants completed an online-based self-report questionnaire that assessed the Big Five personality traits and further person-specific variables (e.g., demographic information). Second, participants nominated up to five people and provided self-reports on relationship-specific information for each person (e.g., relationship type and conflict frequency). All questionnaires were implemented with the software *formr* (Arslan et al., 2020). A detailed account of data collection procedures and study designs of the three original data sets can be found at the corresponding OSF-pages.

### Measures

This study includes two types of data: Person-related variables relating to characteristics of the adolescent individuals and relationship-specific variables relating to the characteristics of their unique relationships with parents and peers. If not specified otherwise, the same variables were measured in all datasets used in Study 1 and Study 2.

#### Person-related variables

The person-related variables were assessed through adolescents’ self-reports and included ratings on adolescents’ personality traits and socio-demographic information.

##### Personality traits

The participants’ level of agreeableness and neuroticism were measured with the German version of the BFI-2 (Danner et al., 2019). Each trait was measured with 12 items. Example items for agreeableness featured statements like “I am respectful, treating others with respect” or “I am compassionate, with a soft heart” while neuroticism included statements such as “I worry a lot” or “I tend to feel depressed, blue”. Participants answered items on a scale from 1 (*strongly disagree*) to 7 (*strongly agree*). In both Studies 1 and 2, scale-based reliability was good for agreeableness (*ω* = 0.87/0.84) and neuroticism (*ω* = 0.90/0.91).

##### Socio-demographic information

The measures included a number of person-specific variables on adolescents’ socio-demographic background that were used as control variables. In both studies, the participants’ age and gender (indicated by identifying themselves as female [0] or male [1]) was included. Additionally, the participants’ original sample (Subsample 1 coded as [0], Subsample 2 coded as [1]) was included as a control variable in Study 1.

#### Relationship-specific variables

The relationship-specific variables were assessed through self-reports from adolescent participants and other-reports from their parents and peers. The variables included perceptions of conflict frequency, the indication of the relationship type, and relationship-specific features (see Tables OS 1 and OS 2 for an overview of other-reports given by parents and peers in Study 1 and 2 and in the respective subsamples of Study 1).

##### Conflict frequency

The frequency of conflicts between the participants and their relationship partners was assessed with the item “Please indicate the frequency of conflicts between you and [name].” Participants answered by selecting one of the following categories: *never*, *rarely*, *sometimes*, *often*, or *almost during each meeting*. In the self-report, participants answered this item for each person that they nominated. In the other-report, relationship partners answered the item once with respect to the participant.

##### Relationship type

The type of relationship for each participant-relationship partner dyad was measured using the item “What is your relationship with [name]?” Relationship partners were categorized as parents if the participants selected the *parents* category in the self-report or if the relationship partners identified the participant as their child in the other-report. People were categorized as peers if the participants or the relationship partners, respectively, selected the category *friend*, *classmate*, *romantic partner*, or *knowing the other person from an association (e.g., sports- or volunteer association)*.^3^ Parents reported knowing the adolescent target person very well (Study 1: *M* = 9.30; Study 2: *M* = 9.06, on a scale from 1–10). Peers also reported knowing the adolescent target person well (Study 1: *M* = 8.47; Study 2: *M* = 8.44).

Relationship-specific features. To account for individual differences in conflict frequency relating to relationship-specific features, contact frequency was assessed with one item. Individuals selected one of the following options: less than once a month, at least once a month, several times a month, once a week, several times a week, or daily. As an indicator of relationship quality, liking was assessed with ratings of how much each participant and parents or peers liked each other on a scale from 1 (not at all) to 10 (very much).

### Data Analysis

To account for the nested data structure (e.g., participants with multiple self-reports of conflict frequency), a path model framework with cluster-robust standard errors was estimated (Cameron & Miller, 2015). To consider the different relationship types (i.e., parents and peers), multigroup models with separate regression paths between the predictor variables (i.e., agreeableness, neuroticism, and the interaction between both traits) and the outcome variables (i.e., perceptions of conflict frequency) for parent and peer relationships were fitted. Agreeableness and neuroticism were centered at the sample mean before analyses (Aiken & West, 1991). All analyses were conducted in *R* (R Core Team, 2023), all models were fitted with the *lavaan* package (Rosseel, 2012). The analytical procedure was identical in Study 1 and Study 2.

In the analytical procedure, predictors were included in the models in a stepwise procedure. A first model included the main effects of agreeableness and neuroticism on self-perceived conflict frequency across relationships (Hypotheses 1 and 2). This model was extended to include the interaction effect between the personality traits (Hypotheses 3a and 3b). Third, a multigroup model was fitted to assess differences between parent and peer relationships. For the full model, this multigroup model was extended to include socio-demographic variables (i.e., adolescent age and gender) and relationship-specific variables (i.e., contact frequency and liking). Figure OS 1 shows the full model. To examine differences between self- and other-perceived conflict frequency (Research Question 2), an identical model set with other-perceived conflict frequency as the dependent variable was estimated. The final multigroup models included adolescents’ age and gender as well as other-reported contact frequency and liking as control variables. In the article, only results of the full multigroup models with control variables are reported.

For the full models, *R*^2^ indicates the goodness of fit. Effects up to *p* < 0.05 are discussed in the text. To test the robustness of the findings, findings were also considered at a stricter significance threshold by adjusting *p* values using the false discovery rate (Benjamini & Hochberg, 1995) in an additional step. Results that did not remain significant after applying this procedure are highlighted. The code and the data necessary to reproduce all results can be retrieved from the OSF (https://osf.io/xmvqd/).

## Results

Table 2 shows the descriptive statistics and intercorrelations of all self- and other-perceived variables for Studies 1 and 2. In both studies, self- and other-perceived conflict frequency was higher in relationships with parents compared to peers. Moreover, adolescents and their relationship partners generally seemed to perceive similar levels of conflict frequency (Study 1: adolescent self-reported conflict frequency: *M* = 2.19, SD = 0.74; other-reported conflict frequency: *M* = 2.33, SD = 0.81; Study 2: adolescent self-reported conflict frequency: *M* = 2.14, SD = 0.63; other-reported conflict frequency: *M* = 2.26, SD = 0.86).

**Table 2:** Means, standard deviations, and correlations of person variables and relationship variables in Study 1 and Study 2

Study 1													
	Self-perceptions		Other-perceptions										
Variable	*M*	SD	*M*	SD	1	2	3	4	5	6	7	8	9
1. Conflict frequency	2.19	0.74	2.33	0.81		**−0.16**	**0.15**	−0.09	**0.13**	**0.20**	−0.02	**0.42**	**0.13**
2. Agreeableness	4.81	0.88	4.98	0.87	**−0.13**		**−0.24**	0.10	**−0.17**	−0.04	**0.16**	0.06	−0.02
3. Neuroticism	4.21	0.96	4.06	1.04	−0.02	**−0.12**		−0.02	**−0.20**	0.08	0.01	−0.02	**−0.13**
4. Age	15.83	1.22	15.89	1.24	−0.08	**0.25**	−0.02		**−0.14**	**−0.13**	0.07	**−0.21**	0.01
5. Gender (1 = male)	0.15	0.36	0.28	0.45	0.05	**−0.12**	**−0.25**	−0.02		−0.03	**−0.17**	0.04	**0.23**
6. Contact frequency	5.33	0.85	5.46	0.77	**0.14**	0.03	−0.00	−0.03	−0.08		**0.30**	**0.34**	−0.03
7. Relationship quality	8.76	1.49	9.39	1.06	**−0.12**	**0.24**	0.05	0.11	−**0.11**	**0.22**		**0.22**	−0.10
8. Relationship type (1 = parent)	0.36	0.40	0.38	0.45	**0.49**	0.02	−0.10	−0.04	0.01	**0.33**	0.10		0.10
9. Sample			0.67	0.47									

Study 2												
1. Conflict frequency	2.14	0.63	2.26	0.89		−0.10	**0.19**	**0.22**	0.12	0.16	**−0.18**	**0.24**
2. Agreeableness	5.05	0.79	5.06	0.77	**−0.26**		**−0.26**	−0.04	−0.11	−0.02	0.09	0.07
3. Neuroticism	3.86	1.08	3.90	1.11	**0.37**	**−0.22**		−0.02	**−0.43**	−0.07	−0.01	−0.03
4. Age	17.71	1.02	17.77	1.00	0.04	−0.08	−0.06		**0.18**	0.12	0.01	−0.00
5. Gender (1 = male)	0.24	0.43	0.22	0.41	**−0.13**	−0.13	**−0.38**	**0.18**		0.08	**−0.24**	−0.02
6. Contact frequency	5.19	0.77	5.35	0.87	0.04	0.03	0.01	**−0.20**	0.09		**0.49**	**0.41**
7. Relationship quality	9.22	0.89	9.56	0.96	**−0.14**	**0.37**	−0.04	−0.13	**−0.15**	**0.27**		**0.25**
8. Relationship type (1 = parent)	0.30	0.30	0.33	0.40	**0.21**	−0.02	**−0.14**	−0.06	0.04	**0.31**	**0.25**	

Self- and other-perceptions refer to relationship-specific variables that were either rated by the adolescent participants or their parents and peers. Personality and sociodemographic variables exclusively represent adolescents’ self-reports. Gender was coded as 0 = female, 1 = male, the Sample variable represents the subsamples in Study 1 (coded as 0 = Subsample 1, 1 = Subsample 2). The Relationship type variable was coded as 0 = peer relationship, 1 = parent relationship. For both Studies 1 and 2, intercorrelations below the diagonal show the links between self-perceived person-related and relationship-specific variables, while intercorrelations above the diagonal illustrate the links between self-perceived person-related variables and other-perceived relationship-specific variables. Correlations significant at *p* < 0.05 are displayed in bold font

### The Role of Agreeableness and Neuroticism for Perceptions of Conflict Frequency

The first hypothesis addressed the main effect of adolescent agreeableness on perceptions of conflict frequency. Table 3 shows the results focusing on self-perceived conflict frequency from Studies 1 and 2, and Table 4 shows the results focusing on other-perceived conflict frequency. In addition, integrating Tables 3 and 4, Table OS 3 provides an overview of the overall result patterns across perceptions and studies. In Study 1, agreeableness played a minor role for self-perceived conflict frequency. Only one significant link emerged between lower agreeableness and increased conflict frequency in parent relationships (*b* = −0.16, *p* = 0.040), but this link proved not robust when applying a stricter significance criterion that accounted for the multiple tested paths within the model (Benjamini & Hochberg, 1995). In Study 2, higher agreeableness was linked to less self-perceived conflict frequency in peer relationships (*b* = −0.14, *p* = 0.005) but not in relationships with parents. In both studies, agreeableness was unrelated to other-perceived conflict frequency in parent or peer relationships (see Table 4). In summary, the overall pattern suggests that adolescents who describe themselves as more agreeable tended to self-report lower conflict frequency, but this pattern was not shared by other-reports of conflict frequency across relationship types.

**Table 3:** Self-perceived conflict frequency predicted by personality traits and relationship-specific variables in Study 1 and Study 2

	Study 1						Study 2					
	Parents (*n* = 246 reports)			Peers (*n* = 525 reports)			Parents (*n* = 196 reports)			Peers (*n* = 483 reports)		
	Est.	SE	*p*	Est.	SE	*p*	Est.	SE	*p*	Est.	SE	*p*
Agreeableness	**−0.16**^**†**^	0.08	0.040	0.01	0.04	0.864	−0.02	0.07	0.765	**−0.14**	0.05	0.010
Neuroticism	0.08	0.06	0.191	−0.04	0.04	0.393	**0.25**	0.06	<0.001	**0.18**	0.04	<0.001
Agreeableness × Neuroticism	0.00	0.06	0.967	0.01	0.03	0.679	0.06	0.06	0.280	−0.05	0.04	0.125
Age	−0.02	0.04	0.647	−0.03	0.03	0.368	0.09	0.08	0.312	0.04	0.04	0.286
Gender	0.11	0.20	0.580	0.01	0.12	0.955	−0.31	0.16	0.050	0.06	0.10	0.554
Contact frequency	0.00	0.13	0.996	**0.07**^**†**^	0.03	0.042	0.10	0.08	0.189	**0.10**	0.03	0.002
Relationship quality	**−0.15**^**†**^	0.06	0.013	**−0.09**^**†**^	0.03	0.007	**−0.27**	0.06	<0.001	−0.06	0.04	0.146
*R*^2^			0.127			0.042			0.238			0.121

In Study 1, results are based on data of *n* = 164 adolescents who provided 246 self-reports on parent relationships and *n* = 247 adolescents who provided 525 self-reports on peer relationships. In Study 2, results are based on data of *n* = 146 adolescents who provided 196 self-reports on parent relationships and *n* = 213 adolescents who provided 483 self-reports on peer relationships. Gender was coded 0 = female and 1 = male. Effects significant at *p* < 0.05 are displayed in bold font

^†^indicates that a *p* value did not remain significant after adjusting for multiple testing (Benjamini & Hochberg, 1995)

**Table 4:** Other-perceived conflict frequency predicted by personality traits and relationship-specific variables in Study 1 and Study 2

	Study 1						Study 2					
	Parents (*n* = 219 reports)			Peers (*n* = 407 reports)			Parents (*n* = 70 reports)			Peers (*n* = 151 reports)		
	Est.	SE	*p*	Est.	SE	*p*	Est.	SE	*p*	Est.	SE	*p*
Agreeableness	−0.11	0.06	0.067	−0.06	0.05	0.193	0.16	0.12	0.188	−0.05	0.09	0.538
Neuroticism	**0.14**	0.05	0.007	**0.09**^**†**^	0.05	0.029	**0.22**	0.07	0.002	0.20	0.10	0.056
Agreeableness × Neuroticism	−0.04	0.05	0.491	0.04	0.05	0.433	0.13	0.09	0.156	0.09	0.08	0.293
Age	−0.02	0.04	0.437	0.03	0.04	0.427	0.07	0.10	0.486	0.17	0.11	0.130
Gender	0.13	0.11	0.267	0.17	0.12	0.144	0.23	0.20	0.260	0.34	0.26	0.176
Contact frequency	**0.24**	0.09	0.008	**0.10**^**†**^	0.04	0.015	0.07	0.12	0.540	0.14	0.08	0.064
Relationship quality	**−0.12**	0.04	0.003	−0.04	0.04	0.264	**−0.63**	0.09	< 0.001	−0.13	0.10	0.195
Sample	0.13	0.13	0.314	**0.21**^**†**^	0.09	0.019						
*R*^2^			0.139			0.067			0.314			0.161

In Study 1, results are based on 175 adolescents whose parents provided 219 other-reports and on 265 adolescents whose peers provided 407 other-reports. Thus, some adolescents received other-reports from both parents and from multiple peers. In Study 2, results are based 61 adolescents whose parents provided 70 other-reports and on 102 adolescents whose peers provided 151 other-reports. Gender was coded 0 = female and 1 = male. Effects significant at *p* < 0.05 are displayed in bold font

^†^indicates that *p* value did not remain significant after adjusting for multiple testing (Benjamini & Hochberg, 1995)

The second hypothesis concerned the role of neuroticism for perceptions of conflict frequency. Unexpectedly, Study 1 revealed no significant link between adolescents’ neuroticism level and their self-perceived conflict frequency in either parent or peer relationships. In contrast, Study 2 showed a positive effect of neuroticism on self-perceived conflict frequency in peer relationships (*b* = 0.18, *p* < 0.001) and parent relationships (*b* = 0.25, *p* < 0.001). Turning to other-perceived conflict frequency, Study 1 revealed that higher neuroticism levels predicted more conflicts reported by relationship partners, especially in relationships with parents (*b* = 0.14, *p* = 0.007). Although the positive link between neuroticism and peer-perceived conflict frequency did not remain significant after applying a more stringent significance criterion, it showed a similar tendency (*b* = 0.09, *p* = 0.029). Parallel results were observed in Study 2 with older adolescents, where neuroticism predicted conflict frequency reported by parents (*b* = 0.22, *p* = 0.002) but the effect was not significant in peer relationships although it showed a similar tendency (*b* = 0.20, *p* = 0.056). Thus, overall, neuroticism emerged as a more consistent predictor of perceived conflict frequency compared to adolescents’ levels of agreeableness. Adolescents who described themselves as more neurotic experienced more frequent conflict across relationships, particularly in the perceptions of parents (see Overview Table OS 3). Regarding the third hypothesis on the interplay of agreeableness and neuroticism, none of the models for self- or other-perceived conflict frequency showed an interaction effect, providing no evidence for a mutual attenuation or a sensitization effect in adolescents’ relationships with parents or peers.

### The Role of Contact Frequency and Relationship Quality for Perceptions of Conflict Frequency

Next to the role of personality, this study examined associations between relationship-specific variables (i.e., contact frequency and relationship quality) and perceptions of conflict frequency in adolescents’ relationships. First, contact frequency was mostly positively associated with conflict frequency: More contact was linked to higher levels of self-perceived conflict in adolescent relationships with peers (*b* = 0.07, *p* = 0.042, note that this effect was not significant at the stricter significance criterion) and also linked to more perceived conflict from the other-perspective of parents (*b* = 0.24, *p* = 0.008) and peers (*b* = 0.10, *p* = 0.015). In parallel to Study 1, results in Study 2 illustrated a positive link between more contact and self-perceived conflict frequency in peer relationships (*b* = 0.10, *p* = 0.002) but no effects emerged for other-perceptions of conflict frequency of parents or peers.

Second, relationship quality was linked to less conflict overall, particularly in relationships with parents: Adolescents who reported more positive relationships with their parents also indicated less conflicts (Study 1: *b* = −0.15, *p* = 0.013, note that this effect was not significant after applying the stricter significance criterion; Study 2: *b* = −0.27, *p* < 0.001). The same was true for other-perceived conflict, where parents reported significantly less conflict when relationship quality was higher (Study 1: *b* = −0.12, *p* = 0.003; Study 2: *b* = −0.63, *p* < 0.001). Summarizing the overall pattern, the quantity captured in contact frequency and the quality represented by relationship quality were differentially related to self- and other-perceptions of conflict frequency across relationship types. Higher contact frequency was somewhat more consistently associated with perceptions of conflict in peer relationships, while relationship quality emerged as a robust predictor of perceptions of conflict frequency in parent relationships.

### Sensitivity Analysis

Two types of sensitivity analyses were performed. First, all models were fitted without the self- and other-reports regarding relationships with romantic partners in the peer category (Study 1: *n* = 44 self-reports and *n* = 16 other-reports excluded; Study 2: *n* = 72 self-reports and *n* = 29 other-reports excluded). Results illustrated that the effects of this stricter exclusion remained largely consistent with the results of the initial approach, with two exceptions in Study 2: Contrary to the main models, there was no main effect of agreeableness on self-perceived conflict frequency in peer relationships. This underlines the overall pattern, suggesting that the role of agreeableness is minor at best for self-perceived conflict frequency. Consistent with the findings from Study 1, the models in Study 2 now displayed a significant main effect of neuroticism on other-perceived conflict frequency in peer relationships, although this effect lost its significance after applying the stricter significance criterion.

As a second sensitivity analysis, models focusing on other-perceived conflict frequency in Study 1 were estimated separately for the two subsamples that were initially merged to increase statistical power. The models for separate subsamples indicated a main effect of agreeableness on other-perceived conflict frequency in parent and peer relationships in Subsample 1 which was not visible in the main analyses using the merged sample or in Subsample 2. Additionally, effects of relationship-specific variables (i.e., contact frequency and relationship quality) emerged inconsistently across samples, but went in the same direction as in the main analysis (see OS Table 4 for details).

## Discussion

Both personality (e.g., Mund & Neyer, 2014) and relationship-specific features (e.g., Adams & Laursen, 2007) are differentially linked to conflict frequency in social relationships. However, an integrative approach examining how these aspects jointly relate to conflict frequency in adolescent relationships is missing from the literature so far. The current research addressed this gap with two studies investigating the roles of the personality traits agreeableness and neuroticism and the relationship-specific features of contact frequency and relationship quality for perceptions of conflict frequency in middle (Study 1) and late (Study 2) adolescence. In doing so, the present research extends previous findings in several ways. First, implementing theoretical notions from personality psychology (Hofstee et al., 1992), it goes beyond the independent effects of agreeableness and neuroticism and investigates how their interplay relates to conflict frequency. Second, by considering parent and peer relationships, it distinguishes between two relevant relationship types that serve different functions in adolescents’ social development. Finally, the two studies test whether personality and relationship effects generalize across perspectives by including both self- and other-perceptions of conflict frequency and relationship-specific features.

Regarding the role of personality, the overall effect pattern illustrates that adolescents’ level of neuroticism was more consistently related to conflict frequency in parent and peer relationships and across self- and other-perceptions than agreeableness. Furthermore, the interplay between agreeableness and neuroticism was not associated with perceptions of conflict frequency in either relationship type. Regarding relationship-specific features, contact frequency and relationship quality were differentially related to perceptions of conflict frequency in parent and peer relationships: More positive relationships with parents were linked to less perceived conflict, while spending more time together was linked to more perceived conflict in relationships with peers.

### The Role of Personality for Conflict Frequency: Neuroticism Takes the Lead

The findings illustrate that neuroticism and agreeableness have different implications for perceptions of conflict frequency in adolescents’ relationships with parents and peers. Specifically, only higher neuroticism was consistently related to higher conflict frequency across relationship types, highlighting the trait’s relevance across interpersonal contexts. The finding that adolescents’ neuroticism was also linked to other-perceived conflict frequency is particularly interesting, since neuroticism is often considered a more internal trait less visible to others (Vazire, 2010). As such, the findings extend previous research showing that neuroticism relates to conflict frequency in adulthood (e.g., Deventer et al., 2019) and highlight that this trait has visible behavioral implications in adolescents’ relationships with parents and peers that are picked up by their relationship partners.

Insights regarding the role of neuroticism in conflict frequency is further refined when comparing result patterns across Studies 1 and 2: Among younger participants in Study 1, adolescents’ neuroticism related to their parents’ and peers’ perceptions of conflict frequency, but not to their self-perceptions. Among older participants in Study 2, in contrast, the link between neuroticism and conflict frequency was similar for self- and other-perceptions, such that adolescents with higher neuroticism perceived more conflict themselves as did their parents and peers. Altogether, these findings suggest that at younger age, adolescents with higher neuroticism do not perceive more conflicts than those with lower neuroticism scores, but their parents and peers do. However, as adolescents get older, individuals with higher neuroticism scores perceive more conflicts in their relationships. This increased perceptual sensitivity associated with neuroticism possibly feeds into a vicious cycle characterized by mutual reinforcement of strain experienced by individuals with higher neuroticism scores and their relationship partners (Jeronimus et al., 2014). Whereas one might suspect that the lack of significant associations between adolescents’ neuroticism and self-perceived conflict frequency in Study 1 might be due to restricted variance in the predictor or outcome variables, standard deviations indicate that this was not the case; variances in Study 1 were similar to those in Study 2. It is important to note, however, that the data of Study 1 were collected during different stages of the COVID-19 pandemic in Germany. Several studies indicate that adolescents were particularly burdened by school closings and contact restrictions (e.g., Rogers et al., 2021). Thus, as an alternative explanation, factors relating to the pandemic situation may have overshadowed the effects of neuroticism on conflict frequency perceived by adolescents themselves, and the differences across findings from Studies 1 and 2 might result from different timings of the data collection despite relying on the same measures and similar study designs. In the future, studies focusing on all phases of adolescence (i.e., early to late adolescence) are needed to clarify the role of personality for perceptions of conflict frequency in this developmental period.

Across relationship types and perceptions, agreeableness was inconsistently related to individual differences in conflict frequency when accounting for neuroticism and relationship-specific variables. In Study 1, agreeableness was associated with lower self-perceived conflict frequency in adolescents’ relationships with parents but not peers In Study 2, in contrast, agreeableness was linked to higher self-perceived conflict frequency in peer but not parent relationships. Finally, in both studies, agreeableness and conflict frequency were not related to each other at all when considering the other-perceptions of parents or peers. Whereas these findings contradict previous studies associating higher agreeableness with less conflict (e.g., Mund & Neyer, 2014), they add to the body of research suggesting that agreeableness and conflict frequency are unrelated (e.g., Jensen-Campbell et al., 2003). As one possible explanation, the weak link between agreeableness may be explained by patterns of normative development (Adams & Laursen, 2001), where certain conflicts, such as those revolving around topics of autonomy and questioning the authority of parents (e.g., Branje, 2018), arise regardless of adolescents’ degree of agreeableness. In addition, agreeableness might be less relevant for the frequency in which conflicts occur, but rather for the way conflicts are handled. This view is corroborated by several studies relating higher agreeableness to more perspective-taking, negotiation, and constructive conflict resolution strategies (e.g., Jensen-Campbell & Graziano, 2001). Taken together, the findings underline the importance of distinguishing between conflict frequency as a quantitative measure of conflict and process-related measures capturing the way conflict is handled. Furthermore, it should be noted that the sensitivity analysis revealed partly divergent associations between agreeableness and conflict frequency across the different subsamples in Study 1. This finding highlights adolescence as a particularly dynamic phase, involving a multitude of developmental forces that may require further attention when studying the link between personality and conflict.

Across models, the findings did not provide evidence for an interplay between agreeableness and neuroticism: The association between adolescents’ neuroticism and higher conflict frequency in their relationships with parents and peers was not buffered by higher levels of agreeableness, providing no support for a mutual attenuation effect. Furthermore, adolescents who were both high in agreeableness and neuroticism were not more successful in preventing conflict, providing no support for a sensitization effect. Thus, previous research on anger and aggression in adulthood suggests that the interplay between agreeableness and neuroticism may affect the way people act during conflicts (e.g., Ode et al., 2008), but this does not seem to generalize to the frequency in which conflicts occur in adolescence. One reason for the lack of an interplay between personality traits could be that the interaction of agreeableness and neuroticism may only manifest in specific interaction contexts or with regard to specific conflict topics, rather than being a general phenomenon across all adolescent relationships.

Overall, the findings suggest that research on conflict frequency should focus on neuroticism, while the potential effects of agreeableness or an interplay between both traits seem non-existent or negligible. At the same time, it should be considered that, on average, adolescents and their relationship partners reported relatively few conflicts, and interaction effects between agreeableness and neuroticism may have been disguised by floor effects (i.e., many participants with values near the lower end of the scale result in low variance). Therefore, future studies should aim to include adolescents who have more conflicts in their relationships and thereby increase variance within the sample.

### The Role of Relationship-Specific Features: Differences between Parent and Peer Relationships

Next to individual differences in conflict frequency relating to adolescents’ personality traits, the current research sheds light on the role of relationship-specific features in this context. Whereas, in line with previous research (Laursen & Collins, 1994), adolescents generally perceived more conflicts with their parents than with their peers, the relationship-specific features that related to higher conflict frequency within each relationship type differed. In adolescent-parent relationships, lower levels of relationship quality (indicated by liking) were linked to more conflict perceived by adolescents and their parents. Conversely, both adolescents and peers reported that frequent contact was linked to more conflicts in their relationships. These differences between parent and peer relationships could be attributed to the distinct characteristics of social relationships with peers and parents.

Previous research has emphasized that relationships with parents are characterized by ambivalence (Fingerman et al., 2008). When living in a shared household, adolescents and their parents spend a lot of time together and conflict may erupt more often due to the few opportunities to avoid contact. As a result, the amount of contact in parent-adolescent relationships may not serve as a reliable indicator of individual differences in conflict frequency but rather the overall quality of the relationship becomes a more important predictor for conflict frequency. In contrast to relationships with parents, peer relationships are characterized by a voluntary and mutual connection (e.g., Laursen & Pursell, 2009). Moreover, many topics that lead to conflicts in adolescents’ relationships with parents, such as adolescents’ rights and responsibilities, tend to be a subject of agreement among peers (Laursen, 1995). Conflicts in peer relationships mainly revolve around interpersonal matters concerning the unique relationship between peers. The current findings might reflect that adolescents dare to address such problematic topics with peers more frequently the more contact they have. Consequently, high contact intensity may offer more opportunities to talk about issues that lead to conflict. Taken together, the findings of this study suggest that structural relationship differences likely contribute to a different frequency of conflicts in parent and peer relationships that is reflected in perceptions of both relationship partners. This way, this study underscores two aspects: first, the importance of investigating conflict frequency in both relationship types separately and, second, the need to account both for differences in contact frequency and relationship quality.

Zooming into the different relationship types, an additional insight from the present research is that adolescents and their relationship partners generally perceived similar mean-levels of conflict frequency. Simply put, adolescents tended to agree with both their parents and peers on how often they experienced conflicts. Aligning with the results, one study focusing on early and mid-adolescence reported considerable overlap between adolescent and peer perceptions of daily conflict (Burk & Laursen, 2005). It is worth noting that discrepant perceptions within relationships can have negative implications for adolescents. For example, discrepancies in parent and adolescent perceptions have been linked to higher levels of perceived stress and depressive symptoms (Human et al. 2016). From this perspective, the findings appear encouraging in that the absolute mean scores of conflict frequency between adolescents and relationship partners were fairly similar.

### Implications

The insights on the role of personality and relationship-specific features on self- and other-perceived conflict frequency have implications for promoting healthy social relationships during adolescence. First, the finding that neuroticism relates to more conflicts with both parents and peers suggests that, in the long term, adolescents with high trait scores might be at risk of experiencing social isolation: They may find it challenging to rely on their peer relationships when facing conflicts with their parents and vice versa. For this reason, healthcare providers and teachers should pay attention to adolescents with high neuroticism and offer social support when needed. Besides inspiring social skills trainings aiming to reduce conflict frequency among adolescents in general (e.g., Vila et al., 2021), research on personality-situation fit points to person-centered approaches through which adolescents with higher neuroticism in particular can be supported. For example, adults with higher neuroticism seem to benefit more from social interactions with friends compared to other interaction partners (Mueller et al., 2019) and from face-to-face compared to digital interactions (Kroencke et al., 2022). Accordingly, interactions with close peers and face-to-face interactions may provide a particularly fruitful environment to train conflict-reducing behavior among adolescents with higher neuroticism to improve social relationships.

Second, heightened rates of conflict are considered a normative feature of parent-adolescent relationships. Fortunately, the current findings revealed only moderate levels of conflict in relationships with parents rather than extreme levels, which are stressful for both adolescents and parents (Laursen & Hafen, 2010). The results suggest that elevated conflict levels (though still in a moderate range) in parent-adolescent relationships are linked to lower perceived relationship quality. Consequently, promoting overall relationship quality might be an important step in preventing conflict rates from escalating in family contexts. Among families that suffer from very frequent conflicts between the adolescent children and their mothers and fathers, parents could take advantage of specific intervention programs to promote the overall quality of the parent-child relationship. Such interventions have been shown to be effective among parents of adolescents showing typical behaviors for this age-group (Shokoohi-Yekta et al., 2015) as well as those who show behavioral problems (Hall & Rose, 1987) and may take place in-person or online (Taylor et al., 2015). In peer relationships, increased time spent together was linked to higher likelihood of conflict in the current work. However, this result does not suggest that adolescents should reduce their contact with peers. On the contrary, intense peer contact offers both the risk of conflict and also the opportunity to balance negative social experiences. Aligning with research emphasizing that conflict in adolescence is not necessarily negative but an essential aspect of social development (Jensen-Campbell et al., 1996), peer relationships characterized by higher conflict frequency likely present a valuable opportunity for individuals to experiment with and develop conflict resolution strategies outside the family context.

### Limitations and Future Directions

While this study has important strengths, such as considering multiple relationship types and self- and partner-perceptions of conflict frequency, it also has several limitations. First, the current work is based on cross-sectional data, which means that it is not possible to draw causal conclusions. Second, although both mid- and late adolescents were included, caution must be taken against making age-differential inferences. To address both limitations, future research should incorporate longitudinal study designs to gain a deeper understanding of how personality traits and relationship features predict conflict frequency and its changes across different phases of adolescence.

Third, the assessment of conflict frequency relied on a one-item measure that lacked a specific time reference, potentially introducing variability in results as adolescents may have interpreted the time frame differently. To enhance precision in future studies, it is recommended to specify the time frame in the instructions (e.g., conflicts in the last days, weeks) to enable a more nuanced analysis of conflict frequency.

Fourth, the findings’ generalizability may be constrained since the samples overrepresented female adolescents (over 75% in both studies) and adolescents attending the highest academic track in the German school system (78–100%). Given that most conflicts in adolescence arise with mothers and same-gender peers (Adams & Laursen, 2001), the findings on conflict frequency in both parent and peer relationships may be largely limited to female-only dyads. In addition, ethnicity may relate to schematic differences in conflict perceptions (Jensen-Campell et al., 1996): The interpretation of behaviors as socially acceptable or conflict-inducing may vary across cultures (Bornstein & Cheah, 2005). For example, adolescents’ increased focus on peer relationships may be normative in Western, predominantly individualistic societies but less encouraged in more kin-oriented societies (Edwards, 1992). Correspondingly, it remains an open question for future research whether the findings on conflict frequency in parent and peer relationships replicate in a sample of adolescents with more diverse cultural backgrounds.

Finally, in Study 1, the COVID-19 pandemic likely impacted the living conditions of adolescents, leading to increased contact with parents due to regulations to stay at home and reduced face-to-face contact with peers. This way, the circumstances of the pandemic might have also impacted the issues that typically lead to conflicts within adolescents’ relationships. This interpretation, however, is complicated by the fact that the experience of the pandemic is confounded with the age of the participants in Study 1. Participants in Study 1 were mostly younger than participants in Study 2. Consequently, it is not clear whether differences between studies may be attributed to COVID-19 as historical context or to age differences. Looking to future research, the current study opens up several promising directions. First, beyond studying conflict in adolescents’ parent relationships, previous research points to generally higher involvement of mothers in adolescents’ education compared to fathers (Phares et al., 2009) and to divergent roles of mothers and fathers in conflicts revolving around the autonomy of their adolescent children (Ravindran et al., 2020). Therefore, the distinction between conflicts with mothers and fathers may add relevant details. Second, moving beyond adolescents’ peer relationships, research focusing on conflict in other types of relationships could provide a more comprehensive understanding of how personality and relationship variables contribute to conflict in adolescence. For example, particularly during adolescence, relationships with siblings or first romantic partners represent developmentally relevant social relationships with unique interpersonal dynamics (Laursen & Bukowski, 1997) and therefore pose compelling research contexts. Third, using experience-sampling methods to study conflict in the everyday lives of adolescents offers another avenue for future research to gain insights into the complexities of conflict dynamics. Specifically, situational characteristics might add to the effects of adolescents’ personality traits on conflict frequency or interact with them. In adolescents’ daily lives, for example, conflicts with parents might occur more often during duty-related situations that involve homework or household tasks, yet the degree to which such situations trigger conflicts likely varies depending on adolescents’ personality (e.g., Rauthmann et al., 2015).

## Conclusion

Previous research on conflict frequency has predominantly focused on links with either personality or relationship-specific features. However, the combined contribution of these factors to perceptions of conflict frequency in adolescence remains largely unexplored. Addressing the need for an integrative investigation, this study illustrates the role of the personality traits agreeableness and neuroticism on the one hand, and the importance of relationship-specific features such as contact frequency and relationship quality for perceptions of conflict frequency on the other. Importantly, this investigation extends prior research by differentiating between perceptions of conflict frequency in parent and peer relationships, as well as considering both the perceptions of adolescents and their relationship partners. Based on this multimethod approach, the findings revealed that adolescents’ neuroticism was an important predictor of conflict frequency across relationships with parents and peers. Regarding relationship-specific features, results highlighted that self- and other-perceived conflict frequency was predicted by poorer relationship quality in adolescent-parent relationships. In contrast, perceived conflict frequency was more consistently linked to contact frequency in adolescent-peer relationships. Overall, the present study suggests that both personality and relationship features are essential to understand conflict in adolescent relationships. The results offer practical insights for parents, suggesting a focus on improving relationship quality as means to reduce conflict with adolescents. Furthermore, it is important to recognize that conflict in adolescence is not always detrimental; particularly in peer relationships characterized by higher contact frequency, conflicts can serve as valuable learning experiences. Finally, interventions including social skill training may prove beneficial in learning how to deal with conflict, especially for adolescents high in neuroticism who are at an increased risk of encountering more strenuous social relationships.

### Supplementary information

Supplementary Information

## Footnotes

1 Statistically, both the mutual attenuation as well as the sensitization effect would be represented by a negative interaction of the two traits. Whereas the interaction would add to the main effects of agreeableness and neuroticism in case of the mutual attenuation effect, it only adds to the main effect of agreeableness in case of the sensitization effect, while the main effect of neuroticism would dissipate.

2 Comparing participants of Study 1 and Study 2 with respect to the study variables showed that participants in Study 2 were older, *t*(519.55) = 22.01, *p* < 0.001, *d* = 0.58, scored higher on agreeableness, *t*(483.21) = 2.78, *p* = 0.006, *d* = 0.09, and lower on neuroticism, *t*(404.15) = −2.94, *p* = 0.003, *d* = −0.11, than participants of Study 1.

3 The plausibility of the self-selected categories in relation to other-reports was assessed by checking the age of the relationship partners within each category and the duration of their acquaintance with the target adolescent.
