# Supplementary material for: The Role of Agreeableness, Neuroticism, and Relationship-Specific Features in Self- and Other-Perceptions of Conflict Frequency in Adolescent Relationships with Parents and Peers
Source: J Youth Adolesc. 2024 Feb 24;53(7):1630–45. doi: 10.1007/s10964-024-01951-6 (PMC11136840; doi:10.1007/s10964-024-01951-6)
Supplement: Supplementary file 1 — Supplementary Information [file 10964_2024_1951_MOESM1_ESM.docx]

**The Role of Agreeableness, Neuroticism, and Relationship-Specific Features in Self- and Other-Perceptions of Conflict Frequency in Adolescent Relationships with Parents and Peers**

**Online Supplement**

**Figure OS 1**


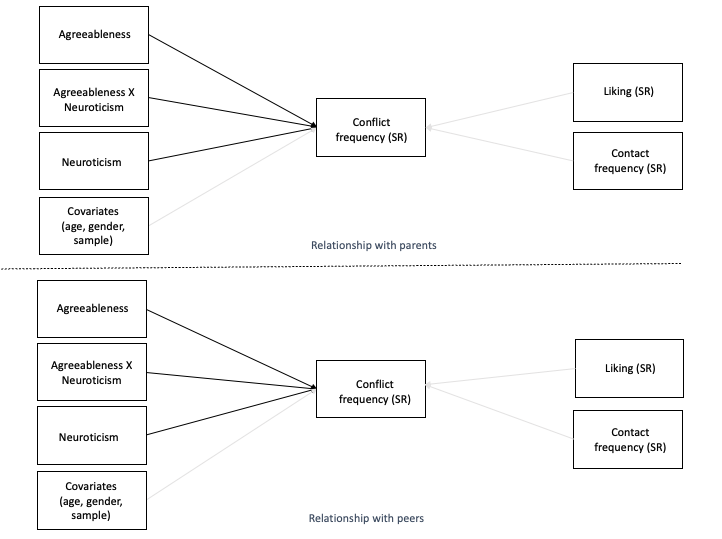
*Multigroup Model Testing the Associations Between Agreeableness, Neuroticism, and the Interplay of the two Personality Traits and Self-Reported Conflict Frequency*

## *Note.* SR = Self-reported. Models with conflict frequency reported by relationship partners have identical set-up. Figure shows a simplified version of the statistical model without covariances.

**Table OS 1**

*Overview of Self- and Other-Reports of Perceived Conflict Frequency in Study 1 and Study 2*

| **Perception** | **Study 1** | |  | **Study 2** | |
| --- | --- | --- | --- | --- | --- |
|  | Parents | Peers |  | Parents | Peers |
| Self-report | 246 | 525 |  | 196 | 483 |
| Other-report | 219 | 407 |  | 70 | 161 |

*Note.* Numbers are based on data from *n* = 571 in Study 1 and *n* = 233 participants in Study 2. Participants could provide more than one self-report and could receive more than one other-report from relationship partners.

**Table OS 2**

*Overview of Self- and Other-Reports of Perceived Conflict Frequency in the Subsamples of Study 1*

| **Perception** | **Subsample 1** | |  | **Subsample 2** | |
| --- | --- | --- | --- | --- | --- |
|  | Parents | Peers |  | Parents | Peers |
| Self-report | 246 | 525 |  | - | - |
| Other-report | 55 | 138 |  | 164 | 269 |

*Note.* Details on both subsamples can be found on OSF (Subsample 1: Wagner et al., 2021; <https://osf.io/r5gjx/>, Subsample 2: Bleckmann & Wagner, 2021; <https://osf.io/w4nmj/>). Comparing participants between the subsamples on person-related key variables, adolescents in Subsample 2 were more likely to be male (*X*^2^(1) = 31.68, *p* < .001) reported higher levels of agreeableness, *t*(550.59) = 2.23, *p* = .026, *d* = 0.19, and slightly lower in neuroticism, *t*(525.19) = -2.96, *p* = .003, *d* = -0.25, compared to adolescents in Subsample 1.There were no mean differences in age.

**Table OS 3**

*Overview of Effect Patterns Across Studies, Self- and Other-Reports, and Relationship Types*

| **Predictor of conflict frequency** | **Corresponding hypothesis** | **Perception** | **Results** | | | | | | |  | **Overall effect pattern** |
| --- | --- | --- | --- | --- | --- | --- | --- | --- | --- | --- | --- |
|  |  |  | Study 1 | | | |  | Study 2 | |  |  |
|  |  |  | Peer | Parent |  | Peer | | | Parent |  |  |
| *Personality-specific* | |  |  |  |  |  | | |  |  |  |
| Agreeableness | H1: Higher agreeableness is associated with self- and other-perceptions of lower conflict frequency. | Self-report | – | NEG^†^ |  | NEG | | | – |  | Agreeableness is negatively related to self-perceived conflict frequency but inconsistently. |
|  |  | Other-report | – | – |  | – | | | – |  | No effect of agreeableness on other-perceived conflict-frequency. |
| Neuroticism | H2: Higher neuroticism is associated with self- and other-perceptions of higher conflict frequency. | Self-report | – | – |  | POS | | | POS |  | Positive effect pattern indicating higher neuroticism is linked to more self-perceived conflict, but inconsistently across relationship types. |
|  |  | Other-report | POS^†^ | POS |  | POS† | | | POS |  | Positive effect pattern linking higher neuroticism to more other-perceived conflict. |
| Interplay between Agreeableness and Neuroticism | H3: Interaction effect between agreeableness and neuroticism on self- and other-perceptions of conflict frequency. | Self-report | – | – |  | – | | | – |  | No interaction effect of agreeableness and neuroticism on self-perceived conflict-frequency. |
|  |  | Other-report | – | – |  | – | | | – |  | No interaction effect of agreeableness and neuroticism on other-perceived conflict-frequency. |
| *Relationship-specific* | |  |  |  |  |  | | |  |  |  |
| Contact frequency |  | Self-report | POS^†^ | – |  | POS | | | – |  | Positive effect pattern linking more contact to more self-perceived conflict in peer relationships. |
|  |  | Other-report | POS^†^ | POS |  | – | | | – |  | Positive effect pattern linking more contact to more other-perceived conflict, only in Study 1. |
| Relationship quality |  | Self-report | NEG^†^ | NEG^†^ |  | – | | | NEG |  | Negative effect pattern linking higher relationship quality to less self-perceived conflict across relationships. |
|  |  | Other-report | – | NEG | | |  | – | NEG |  | Negative effect pattern linking higher relationship quality to less other-perceived conflict in parent relationships. |

*Note.* NEG = negative effect in multigroup model, POS = positive effect in multigroup model. **^†^** indicates that *p*-value did not remain significant after adjusting for multiple testing (Benjamini & Hochberg, 1995).

**Table OS 4**

*Other-Perceived Conflict Frequency Predicted by Personality Traits and Relationship-Specific Variables the Subsamples of Study 1*

|  | **Subsample 1** | | | | | | |  | **Subsample 2** | | | | | | | | |
| --- | --- | --- | --- | --- | --- | --- | --- | --- | --- | --- | --- | --- | --- | --- | --- | --- | --- |
|  | Parents (*n* = 55 reports) | | |  | Peers (*n* = 138 reports) | | | | |  | Parents (*n =* 164 reports) | | |  | Peers (*n =* 269 reports) | | |
|  | *Est.* | *SE* | *p* |  | *Est.* | *SE* | *p* | | |  | *Est.* | *SE* | *p* |  | *Est.* | *SE* | *p* |
| Agreeableness | **-0.51** | 0.14 | < .001 |  | **-0.19^†^** | 0.09 | .040 | | |  | -0.00 | 0.07 | .995 |  | -0.03 | 0.07 | .679 |
| Neuroticism | -0.12 | 0.12 | .312 |  | 0.07 | 0.06 | .251 | | |  | **0.20** | 0.06 | .001 |  | 0.10 | 0.06 | .096 |
| Agreeableness × Neuroticism | -0.04 | 0.50 | .617 |  | 0.04 | 0.06 | .498 | | |  | -0.06 | 0.06 | .352 |  | 0.04 | 0.06 | .487 |
| Age | -0.02 | 0.10 | .827 |  | 0.09 | 0.08 | .250 | | |  | -0.02 | 0.05 | .738 |  | 0.01 | 0.05 | .858 |
| Gender | -0.38 | 0.49 | .430 |  | -0.10 | 0.25 | .679 | | |  | 0.13 | 0.12 | .277 |  | 0.22 | 0.13 | .076 |
| Contact frequency | -0.58 | 0.40 | .140 |  | 0.07 | 0.05 | .117 | | |  | **0.26** | 0.09 | .005 |  | **0.12^†^** | 0.06 | .038 |
| Relationship quality | **-0.58^†^** | 0.29 | .041 |  | -0.08 | 0.07 | .289 | | |  | **-0.13** | 0.04 | .001 |  | -0.04 | 0.05 | .439 |
| *R*^2^ | .284 | | |  | .079 | | | | |  | .160 | | |  | .054 | | |

*Note.* In Subsample 1, results are based on 46 adolescents whose parents provided 55 other-reports and on 91 adolescents whose peers provided 138 other-reports. Thus, some adolescents received other-reports from both parents and from multiple peers. In Subsample 2, results are based on 129 adolescents whose parents provided 164 other-reports and on 174 adolescents whose peers provided 269 other-reports. Gender was coded 0 = female and 1 = male. Effects significant at *p* < .05 are displayed in bold font. **^†^** indicates that *p*-value did not remain significant after adjusting for multiple testing (Benjamini & Hochberg, 1995).
